# Extremely lethal and hypervirulent *Mycobacterium tuberculosis* strain cluster emerging in Far East, Russia

**DOI:** 10.1080/22221751.2021.1967704

**Published:** 2021-08-22

**Authors:** Tatiana Vinogradova, Marine Dogonadze, Natalia Zabolotnykh, Maria Badleeva, Irina Yarusova, Anna Vyazovaya, Alena Gerasimova, Svetlana Zhdanova, Maria Vitovskaya, Natalia Solovieva, Oksana Pasechnik, Oleg Ogarkov, Igor Mokrousov

**Affiliations:** a Laboratory of Molecular Epidemiology and Evolutionary Genetics, St. Petersburg Pasteur Institute, St. Petersburg, Russia; b Laboratory of Experimental Tuberculosis and New Medical Technologies, St. Petersburg Research Institute of Phthisiopulmonology, St. Petersburg, Russia; c Bacteriology Laboratory, St. Petersburg Research Institute of Phthisiopulmonology, St. Petersburg, Russia; d Department of Infectious Diseases, Buryat State University, Ulan-Ude, Buryatia, Russia; e Bacteriology Laboratory, Clinical Anti-tuberculosis Dispensary, Omsk, Russia; f Department of Epidemiology and Microbiology, Scientific Centre of the Family Health and Human Reproduction Problems, Irkutsk, Russia; g Department of Public Health, Omsk State Medical University, Omsk, Russia

**Keywords:** *Mycobacterium tuberculosis*, virulence, mouse model, Beijing genotype, epidemic

## Abstract

*Mycobacterium tuberculosis* strains of the early ancient sublineage of the Beijing genotype are mostly drug susceptible and mainly circulate in East Asia. We have recently discovered two clusters of this sublineage emerging in the Asian part of Russia (VNTR-defined 1071-32 and 14717-15 types) and, to our surprise, both were strongly MDR/XDR-associated. Here, we evaluated their pathogenic features. The clinical isolates and reference laboratory strain H37Rv were investigated in the C57BL/6 mouse model to assess their virulence and lethality properties. The BACTEC MGIT 960 system was used to study the in vitro growth characteristics. In the murine model, strains 396 (14717-15-cluster, from Buryatia, Far East) and 6691 (1071-32-cluster, from Omsk, Siberia) demonstrated contrasting properties. The 396-infected group had significantly higher mortality, more weight loss, higher bacterial burden, and more severe lung pathology. Furthermore, compared to the previously published data on other Russian epidemic Beijing strains (B0/W148, CAO, Central Asian Russian), strain 396 demonstrated the highest mortality. Under the in vitro growth experiment, cluster 14717-15 isolates had significantly shorter lag-phase. To conclude, low-virulent MDR strain 6691 belongs to the Beijing 1071-32-cluster widespread across FSU countries but at low prevalence. This corresponds to common expectation that multiple drug resistance mutations reduce fitness and virulence. In contrast, highly lethal and hypervirulent MDR strain 396 represents an intriguing Beijing 14717-15 cluster predominant only in Buryatia, Far East (16%), sporadically found beyond it, but not forming clusters of transmission. Further in-depth study of this most virulent Russian Beijing cluster is warranted.

## Introduction

*Mycobacterium tuberculosis* complex strains are capable of infecting different mammalian species with variable symptoms and overall pathogenesis. Mice are the most commonly used in experimental models of *M. tuberculosis* infection to observe variation in virulence of different strains. Experimental animal models of tuberculosis (TB), particularly mouse models, take advantage of a uniform immune response within the host. The animal studies have shown significant differences among mycobacterial strains, and although their findings cannot be directly extrapolated to human infection due to differences in the pathogenesis of TB in humans and mice, they have helped to improve understanding of the pathogenesis and presentation of the disease [1,2].

*M. tuberculosis* is subdivided into several phylogenetic lineages and the Beijing genotype (a major part of the East-Asian lineage or Lineage 2) is one of those most studied. It is subdivided into more heterogeneous ancestral (also termed “ancient”) and evolutionarily younger modern sublineages. Initially, these sublineages were defined as typical and atypical based on IS*6110*-RFLP typing and some other markers [3] and were further validated by robust SNPs and unique deletions [4,5] (Figure S1).

Previous studies demonstrated the association of different sublineages of *M. tuberculosis* with distinct pathological and clinical phenotypes that influence the transmissibility of the particular strains in human populations. Diversity of virulence in a mouse model was previously demonstrated for different *M. tuberculosis* strains and lineages [6]. However, the phylogenetic lineages are heterogeneous and studies involving a single strain per lineage may not take into account an intra-lineage diversity. For example, a study of the H37Rv reference strain and single Beijing (Lineage 2) and EAI (Lineage 1) isolates demonstrated an increased virulence of two clinical isolates compared to H37Rv due to different systemic immune response [7]. However, reduced virulence (100-fold lower bacterial load, lower numbers of cellular infiltrates and smaller granulomatous lesions) was shown for EAI compared to Beijing isolates in another study [8]. Since EAI is ancestral and most heterogeneous among *M. tuberculosis sensu stricto* lineages [9], the EAI isolates in the above studies likely represented its different and distant sublineages. The Beijing genotype was long believed to be genetically homogeneous but studies demonstrated its diversity (Figure S1) and clinical or epidemiological relevance of its certain subtypes. Within the Beijing genotype, its ancient and modern strains differ in their virulence that also partly depends on the strain origin. A study in Brazil and Mozambique, countries with a low prevalence of Beijing strains, showed that strains of the modern Beijing sublineage were mostly highly pathogenic, unlike intermediate or low virulent isolates of the ancient sublineage [10].

The modern Beijing sublineage is globally spread and includes several well-known epidemic or endemic genetic clusters. In contrast, strains of the early ancient sublineage of the Beijing genotype are rarely encountered outside East Asia. In our recent study, we demonstrated a certain increase in prevalence of these strains in Siberia, Russia, furthermore accompanied with a strong association with MDR/XDR. One of the clusters, Mlva 1071-32 was most prevalent (7%) in Omsk, Siberia, and sporadically described in different parts of Russia since the mid-1990s. The second cluster Mlva 14717-15 was found only in Asian Russia where it showed some gradient from 2.6% in Omsk [11] to 16% in Buryatia, Far East [12] (Figure S2).

Previously, the C57BL/6 resistant mice infected with highly virulent *M. tuberculosis* strain were shown as a TB model reproducing an exacerbated inflammatory response in a resistant host to hypervirulent mycobacteria, leading to irreversible necrotic lung lesions [13]. Properties of the Beijing strains were previously studied in this model, including ancient and modern sublineages from different countries [10,14]. However, only modern Beijing sublineage strains from Russia were analysed in those studies. In this study, we aimed to investigate the virulence properties of the *M. tuberculosis* strains of the recently described and MDR-associated ancient Beijing clusters from Russia in the C57BL/6 mouse model. We also compared an enlarged panel of these strains using an in vitro growth model.

## Materials and methods

### M. tuberculosis in vitro growth rate experiment

The framework of the study design is shown in Supplementary Figure S3. Two lines of experiments were conducted to investigate the pathogenic properties of the *M. tuberculosis* isolates in the in vitro growth and in vivo murine models.

The phenotypic property of the growth rate was assessed in vitro for 14 *M. tuberculosis* strains. The strains were recovered from the respiratory material of patients with infiltrative or fibrous-cavernous pulmonary TB. Based on spoligotyping and genotyping of the cluster-specific markers (RD181, NTF locus, *mutT2* codon 58 and *mutT4* codon 48), they belonged to the early ancient sublineage of the Beijing genotype with wild type of *mutT2* codon 58 and *mutT4* codon 48. Based on 24-MIRU-VNTR typing and comparison to the MIRU-VNTRplus.org online tool, 7 isolates belonged to the 14717-15-cluster (intact RD181) and 7 isolates belonged to the 1071-32-cluster (deleted RD181) (Figure S1).

VNTR typing and spoligotyping and detection of other molecular markers were performed as described previously [11,15].

Drug susceptibility testing of anti-tuberculosis drugs was carried out using the method of absolute concentrations (Order No. 109 of the Ministry of Health of the Russian Federation) and/or using the automated system BACTEC MGIT 960. The growth characteristics of the studied strains were evaluated in vitro using the automated BACTEC MGIT system.

The studied strains were stored as a thick suspension in physiological saline buffer supplemented with 15% glycerol at −80°C. Three weeks prior to the experiment, the suspension was thawed and plated onto the Loewenstein-Jensen medium. A small amount of the grown bacterial culture was transferred from the medium to a test tube with 3 mm glass beads. The *M. tuberculosis* culture was vortexed for 30–40 s, and 5 ml of Middlebrook 7H9 broth (Becton Dickinson) supplemented with 0.02% Tween 80 was added. The mix was centrifuged at 1000 rpm for 10 min, the supernatant (top 2 cm) was adjusted to 1 McFarland unit with DenciLametr densitometer (approximately 3 × 10^8^ bacteria/ml). 200 μL of the resulting suspension were transferred into MGIT tube with the OADC growth additive and placed in the automated BACTEC MGIT 960 system. After the growth curve reached a plateau, the numerical data of the system were recorded and processed. The following parameters were determined (in hours): (1) lag-phase, (2) reaching the stationary phase (plateau), (3) time of the maximum hourly increase in fluorescence (i.e. compared to the previous hour). A MATLAB program was used to calculate the r coefficient characterizing the growth curve.

For each of the studied strains, three experiments were performed in three replicates for each experiment (a total of 9 points per strain). Seven strains per cluster were included to assess inter- and intra-cluster diversity. For each strain, the average value was calculated for each of the growth parameters in each of the three series of experiments. Differences in growth indicators of strains of the two clusters were assessed using the Mann–Whitney U test.

### M. tuberculosis strains used for in vivo experiment

The mouse model study included three *M. tuberculosis* strains - two clinical isolates (selected among those tested in the in vitro growth experiment) and one reference laboratory strain H37Rv. The reference strain H37Rv was received from the Collection of the Scientific Center for the Expertise of Medicinal Products, Moscow, Russia (and initially received from the Institute of Hygiene and Epidemiology, Prague, Czech Republic). The clinical *M. tuberculosis* strains 396 and 6691 belonged to the early ancient sublineage of the Beijing genotype, i.e. they had RD105 and RD207 deletion, and wild type alleles of the *mutT2* codon 58 and *mutT4* codon 48. Strain 396 had intact locus RD181, spoligotype SIT269 and was characterized by Mlva profile 14717-15. Strain 6691 had deleted RD181, spoligotype SIT1 and was characterized by Mlva profile 1071-32 (Figure S1). Both clinical isolates were multidrug-resistant.

The strains were cultured on Loewenstein-Jensen medium (Becton Dickinson, USA) and after 3 weeks of incubation at +35°C, the culture was suspended in a physiological solution with 15% glycerol, placed in cryovials, frozen and stored at −80°C. Three weeks before the experiment, all strains were recultured on Loewenstein-Jensen medium.

### Experimental animals

All experimental procedures were carried out in accordance with National guidelines [16]. In total, 210 C57BL/6 male mice (weight 16–18 g) were used in all experiments. The mice were obtained from the Andreevka laboratory animal nursery (Moscow region, Russia).

The animals were kept under the conditions of a certified animal facility at the St. Petersburg Research Institute of Phthisiopulmonology using NexGen Mouse IVC Cage & Rack system with built-in ventilation and air conditioning system. Before the start of the study, laboratory animals were quarantined for 14 days. The body weight was monitored weekly using an Adventurer™ electronic balance (OHAUS Corporation, USA). The criteria for inclusion of the animals in the experiment were as follows: positive dynamics of the body weight of animals during the quarantine period and the absence of visible symptoms of the disease.

All procedures with animals were reviewed and approved by the local Ethical Committee of the St. Petersburg Research Institute of Phthisiopulmonology.

### Animal study design

A mycobacterial suspension for infecting mice was prepared ex tempore from three-week-old strains. The infecting dose was 10^6^ CFU in 0.2 ml of saline buffer per mouse. A suspension of mycobacteria was inoculated into the lateral tail vein of the animals.

The animals were divided into two series aimed to study the virulence of strains and survival of animals infected with mycobacteria.

In the first series, 144 mice were observed, 48 mice for each of the 3 studied *M. tuberculosis* strains. The animals were euthanized in groups of 6 mice at days 1, 3, 7, 14, 21, 28, 60, and 120 p.i. Next, an autopsy and a sterile sampling of the lungs and spleen were performed for further culturing of mycobacteria. Lungs and spleen were weighed for calculation of the weight coefficients. Lungs and spleens of the mice were aseptically removed. The weight of each lung was calculated and the gross anatomic picture was taken in order to document the extent of the lesions.

The second series consisted of 60 mice, i.e. 20 per strain. The natural death of animals was recorded during 200 days after infection. The animals that died during the study were subjected to an autopsy with examination of the internal organs.

The body weight of the model animals in both experimental series was monitored once a week. The organ weight coefficients for the lungs and spleen were calculated based on the ratio of the organ weight to the body weight of the animal. The index of lung damage was established based on the combined estimation of exudative and productive changes expressed in conventional units (Table S1) [17].

The homogenized organs (0.1 g of lungs and whole spleen) were cultured on Loewenstein-Jensen medium (Becton Dickinson, USA) by using the method of serial dilutions. The number of grown colonies of *M. tuberculosis* was counted after 4 weeks of incubation at +37°C. The growth of mycobacteria in cultures of lung homogenates was recalculated per organ’s weight. Results were expressed as log_10_ CFU per organ.

For statistical analysis, Microsoft Excel 2013 and Statistica 13.0 programs were used. The significance of differences was assessed by the Student’s *t*-test and was considered significant at *p *< 0.05. Lethality analysis of the model animals was performed using the Kaplan-Meier method.

## Results

### Growth rate analysis in vitro

This experiment included 7 isolates per VNTR cluster. A significant intra-cluster diversity of the growth parameters was observed (Table 1, Table S2). According to the integral growth parameter – the coefficient r, characterizing the features of the growth curve as a whole, according to the U criterion, the groups did not differ, with a weak trend of the Beijing 1071-32-cluster strains to the slower growth. However, the studied groups differed in the duration of the lag phase and the point of maximum hourly increase in fluorescence (*P* = 0.05). The strains of the Beijing 1071-32 cluster had a longer lag phase and had a maximum hourly increase in fluorescence at a later time-point (Table 1).

**Table 1.** Comparison of growth characteristics of the two clusters.

Cluster	Lag-phase, hs	Lag + Log phases, hs	Time of maximum hourly increase of fluorescence compared to the previous time point, hs	Log-phase, hs	Growth rate coefficient r
14717-15	81.7	280.7	146.9	199.0	0.03927
1071-32	116.5	305.1	188.4	188.7	0.03397
Mann–Whitney U-test					
Uemp	9	18	9	24	12
Ucr	11	11	11	11	11
*p*	0.05	>0.05	0.05	>0.05	>0.05

Notes: The average values of the data obtained in three experiments are given. The duration of the lag phase was determined as period between the time of inoculation and time of appearance of fluorescence.

### Virulence of M. tuberculosis isolates in the C57BL/6 mouse model

To characterize the severity of the disease caused by *M. tuberculosis* strains, we quantified the bacterial load in the lungs and spleen of mice infected by strains from each VNTR-cluster and measured body weight loss and organ weight-based parameters in infected animals as an indicator of morbidity and performed a visual assessment of the lung damage at days 1, 3, 7, 14, 21, 28, 60, and 120 p.i.

#### Organ weight changes

**Figure 1.**
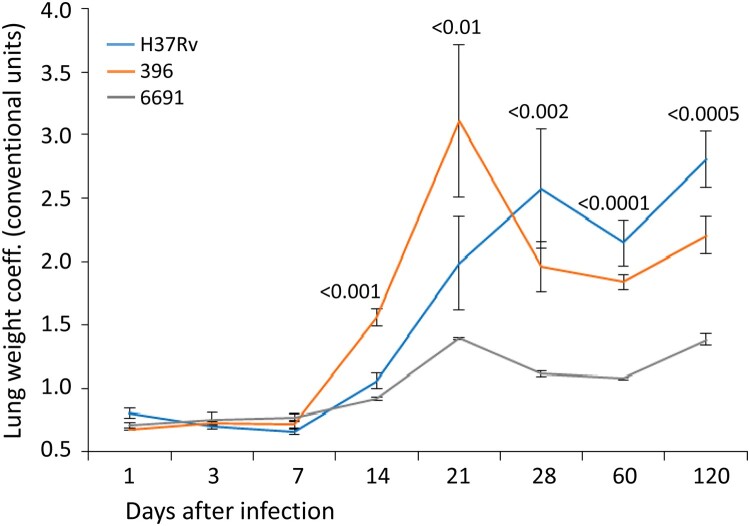
Lung weight coefficient of mice infected with *M. tuberculosis* strains determined at different time points. The same colour is used to depict three studies in all figures. Data represent means plus standard deviations (SD) (error bars). Significant differences between two clinical isolates at particular time points are indicated by *P* values.

**Figure 2.**
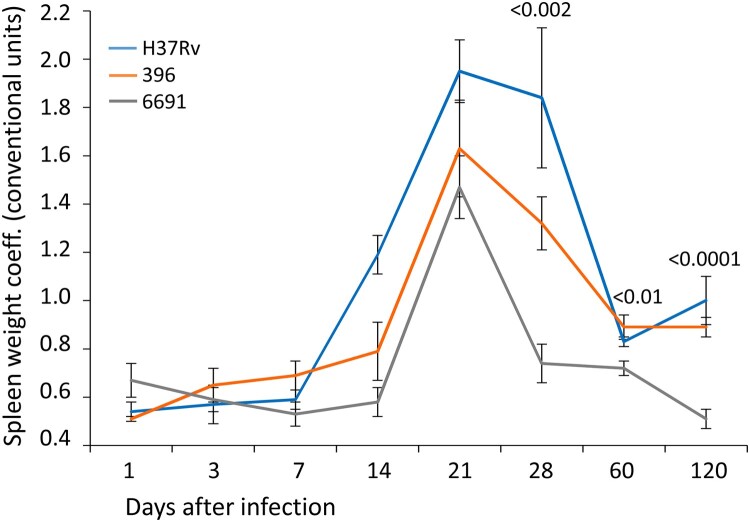
Spleen weight coefficient of mice infected with *M. tuberculosis* strains determined at different time points. Data represent means plus standard deviations (SD) (error bars). Significant differences between two clinical isolates at particular time points are indicated by *P* values.

The analysis of the lung and spleen weight parameters did not show a difference between the three strains at days 1–7 p.i. (Figures 1 and 2).

The lung weight coefficient reflects the development of the specific inflammatory changes in the lung tissue. Its significant increase (*p* < 0.001) was recorded on the days 14–21 in all three groups of mice and was most pronounced (more than twice) in mice infected with strain 396. The lung weight coefficient was significantly lower in mice infected with strain 6691 compared to the two other groups of animals (*р* < 0.001) (Figure 1). This also correlated with lung damage and lung bacterial load, as well as spleen weight coefficient in strain 396 that also peaked at day 21 p.i. (see below).

An overall gradual increase in the lung weight coefficient was observed during the subsequent follow-up period in the mice infected with H37Rv. The highest value was recorded in the group infected with strain 396 at day 21 p.i., which further sharply decreased and was significantly lower than in the control group by day 120 p.i. (*p* < 0.05). The lung weight coefficient values in the group infected with strain 6691 showed some increase at day 21 p.i. but were significantly lower compared to the other two groups at all time points.

The spleen weight coefficient started to increase significantly in the H37Rv- and 396-infected groups since day 14 p.i., and in the 6691-infected group between days 14 and 21 (Figure 2). The increase was abrupt in all groups and was the highest at day 21 p.i. (*p* < 0.001). The lowest values of this indicator at almost all time points were in the mice infected with strain 6691 and were significantly lower than in the H37Rv- and 396-infected mice (*p* < 0.05 and *p* < 0.0001, respectively). The highest values were observed in animals infected with H37Rv at days 14–28.

#### Lung pathology

Based on the visual assessment of lung damage, almost all euthanized mice in all three groups had single airless foci already on the day 3 p.i., indicating the development of the exudative component of specific inflammation and a significant increase of the lung damage index in all three groups of mice compared to the day 1 p.i. (*P* < 0.001). Further progression of the specific process in the lungs (area of exudative changes) since day 7 followed different courses in the studied groups. In the mice infected with strain 6691, an insignificant exudation persisted until day 60, whereas airless areas were observed in one-half to two-thirds of lungs in mice infected with H37Rv and 396 since day 21. Complete airlessness of the lungs was noted only in mice infected with strain 396 and was found in 50% of these mice at day 120.

**Figure 3.**
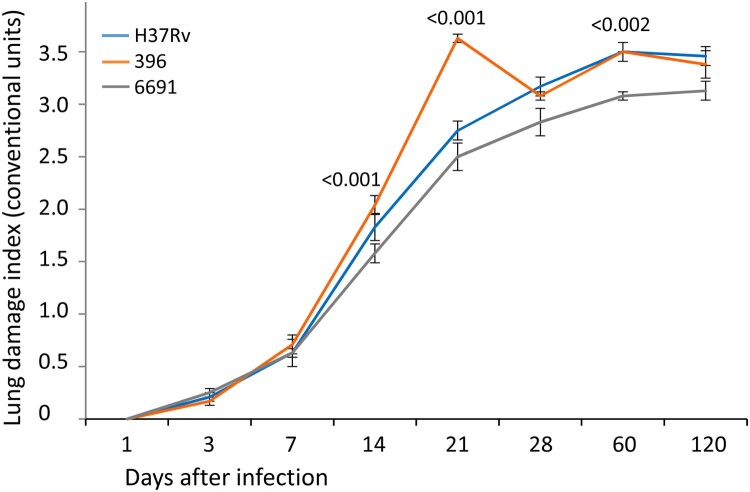
Lung pathology scores of mice infected with *M. tuberculosis* strains determined at different time points. Data represent means plus standard deviations (SD) (error bars). Significant differences between two clinical isolates at particular time points are indicated by *P* values.

Since day 7 p.i., the index of lung damage increased significantly in all groups of mice (*р* < 0.02 to *р* < 0.001) due to the appearance of single submiliary foci of productive inflammation, and most often they were recorded in mice infected with strain 396. Multiple submiliary and miliary productive foci were identified since day 14, and also most often in mice infected with strain 396. The multiple miliary foci started to merge in mice infected with strain 396 since day 21 p.i., compared to day 28 p.i. in mice infected with strain H37Rv. In contrast, this feature was not detected in mice infected with strain 6691 even on day 120 p.i. The highest value of the lung damage index and significantly higher than in other groups was recorded in mice infected with strain 396 on day 21 p.i., while the lowest and often significantly lower values were in mice infected with strain 6691 (*p* < 0.02 to *p* < 0.001). At the end of the observation period (120 days after infection), the index of lung damage was similar in mice infected with H37Rv and 396 (Figure 3). At the same time, foci of necrosis in the H37Rv group were not observed and were found only in 1 of 6 mice in the strain 396-infected mice.

#### Bacterial load

**Figure 4.**
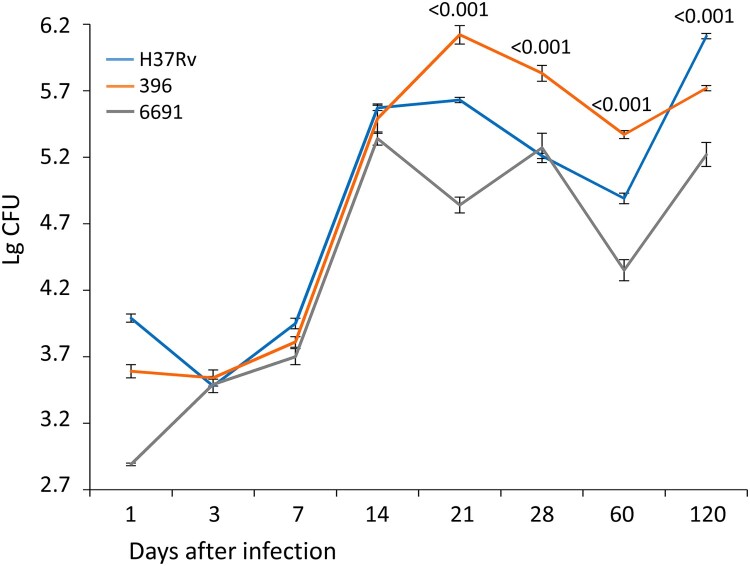
Bacterial load in the lungs of mice infected with *M. tuberculosis* strains determined at different time points. Data represent means plus standard deviations (SD) (error bars). Significant differences between two clinical isolates at particular time points are indicated by *P* values.

**Figure 5.**
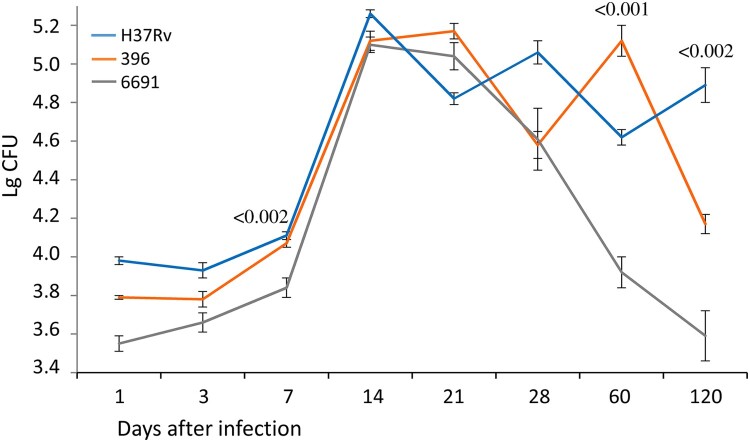
Bacterial load in the spleen of mice infected with *M. tuberculosis* strains determined at different time points. Data represent means plus standard deviations (SD) (error bars). Significant differences between two clinical isolates at particular time points are indicated by *P* values.

The bacterial load of the lungs and spleen was used as an indicator of the severity of the course of TB infection. The bacteriological study confirmed the above observations with regard to the differential virulence of the studied strains (Figures 4 and 5). The bacterial load in the lungs and spleen was significantly lower in mice infected with strain 6691 than in the other two groups during the experiment (*р* < 0.01 to *р* < 0.001).

The bacterial load in the lungs of mice infected with strain 396 increased much faster than in the control group and reached its maximum values by day 21 p.i., followed by a significant decrease (*р* < 0.02) but remained high enough during all 120 days of observation. In the H37Rv infected mice, the bacterial load of lungs increased gradually and the maximum CFU level was observed only by day 120, significantly exceeding this value in the strain 396-infected group (*р* < 0.001) (Figure 4).

Comparison of the lung bacterial load and pathology changes in the three groups of mice revealed a positive correlation for all strains (Figure S4). However, although the correlation coefficients did not differ significantly between the strains, we note the strongest correlation for the strain 396 (0.98 compared to 0.86–0.87 in 6691 and H37Rv).

The spleen, as well as the lungs and the liver, belongs to the organs of predominant accumulation of *M. tuberculosis*. The maximum bacterial load in the spleen of mice infected with strain 396 was recorded on day 21 p.i. followed by a gradual decrease, reaching a significantly lower CFU value compared to the H37Rv group on day 120 (*p* < 0.001). The lowest values of this indicator were in the strain 6691-infected mice. The highest values of the bacterial load in mice infected with strain H37Rv were recorded on day 14 p.i. followed by a significant decrease.

#### Survival of C57BL/6 mice infected with M. tuberculosis strains

**Figure 6.**
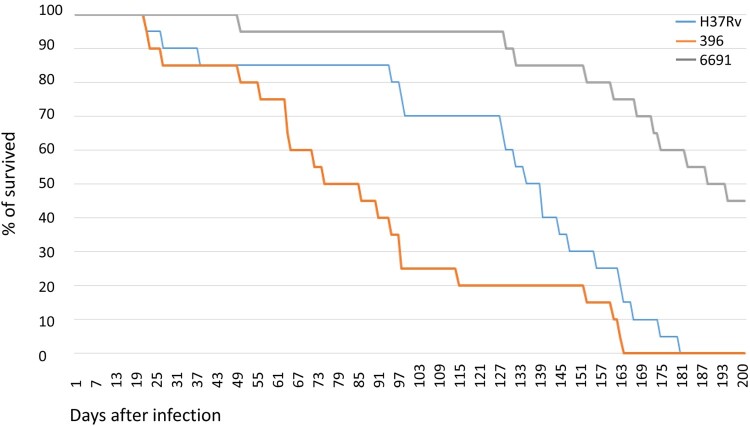
Survival of mice after infection with *M. tuberculosis* strains. Strain 6691 from Omsk belongs to 1071-32- cluster (RD181 deleted), strain 396 from Buryatia belongs to 14717-15-cluster (RD181 intact).

The strains caused different lethality patterns in the infected groups of animals (Figure 6). The death of mice due to disseminated tuberculous lung lesions was first recorded on day 22 p.i. in the mice infected with H37Rv and 396, but in the latter group the death rate increased more rapidly from day 50 to day 100 when the three groups demonstrated the most remarkable difference (Figure 6). For example, while the death rate in the H37Rv and 396-infected groups was identical on the day 49 (15%), 60% of mice died in the 396 group on day 94 (compared to 15% in the H37Rv group and 5% in the 6691 group), and 80% died on day 115 (compared to 30% in the H37Rv and 5% in the 6691 groups). Finally, all mice succumbed in the 396 and H37Rv infected groups on days 164 and 181, respectively.

The lowest mortality rates were observed in the group infected with strain 6691, where animals started to die only after 50 days of the experiment, and 55% of them died by day 200 p.i.

Analysis of the body weight of animals in the lethality experiment showed that the highest mean weight values, indicating the more favourable course of infection, were observed in mice infected with strain 6691, and the lowest – in mice infected with strain 396 (before the death of most of the mice in this group) (not shown). Regarding dynamic changes, the mean weight of mice infected with strains H37Rv and 6691 decreased since the 13th week, reflecting the development of a pronounced intoxication. A slight increase in the mean weight of survived animals in the 396-infected group since the 16th week can be explained by the death of 75% of mice with a severe course of the disease by that time.

## Discussion

### Correlation of the virulence and survival characteristics

This study was undertaken to assess the biological properties of two *M. tuberculosis* strain clusters emerging in the Asian part of Russia. These strains have recently attracted attention due to their unexpectedly strong association with MDR/XDR and local population increase [11,12,15]. We studied particular features of the in vitro growth of these strains and used a model of pulmonary infection of C57BL/6 mice to assess their virulence. The obtained results demonstrate remarkably contrasting pathogenic features of these two clusters, in terms of lethality and virulence, that likely mirror different modes of mycobacterial adaptation to the human host.

Our results show that the studied strains varied in the mice experiment in their lethality measured by survival curves, and their virulence assessed by lung pathology, bacillary load, and lung and spleen weight coefficients measured at different time points after infection. In the previous studies, a correlation between the key features – bacterial load in lungs, lung damage, lung weight coefficient, body weight, and survival – was frequently demonstrated. Measures of lung and body weights concordantly indicated that the *M. bovis* infected mice had higher mortality, more weight loss, higher bacterial burden, and more severe lesions in lungs than *M. tuberculosis* and *M. avium* infected mice [18]. A study of the diverse collection of *M. tuberculosis* complex isolates showed that a severe pathological response correlates with high mortality and high CFU counts in lungs [6] but the same study showed an exceptional case of *M. canettii* with a minimal pathology score but relatively high CFU-counts. Since clinical tuberculosis due to *M. canettii* is rare, this discrepancy between histopathology and CFU counts highlights that it is not the bacterial load per se but the host immune response that determines the clinical effects of infection. Ribeiro et al. [10] noted that the higher virulence of modern Beijing strains was based on their ability to induce severe lung pathology, rather than on increased bacterial growth in the lungs. Similarly, in another Russian study, the bacterial load after infection with Beijing clustered strains was lower than that of H37Rv. B0/W148 cluster, known as transmissible, had a similar level of virulence as the non-clustered strain of the Central Asian/Russian clade [14]. However, in our study, we did demonstrate a correlation between the mortality rates of infected mice and the severity of pathological changes in the lung tissue, as well as the bacterial load in the lungs and spleen.

The strains included in this study belong to the ancient sublineage of the *M. tuberculosis* Beijing genotype. This sublineage makes a main component of the *M. tuberculosis* population structure in Japan and Korea. However, these two Russian clusters appear specific for the Asian part of Russia. Both could have descended from East Asian strains but circumstances and time of their likely Russian origin are elusive. A WGS study of Russian and East Asian Beijing strains [15] did not find a link between the Russian Beijing 1071-32 cluster (low-virulent strain 6691 in our study) and any location outside Russia. In contrast, a smaller cluster Beijing 14717-15 (hypervirulent strain 396 in our study) was located within the larger branch that included isolates from Korea. Previous virulence studies of the Beijing genotype focused on the strains of the globally spread modern sublineage. Their epidemic prevalence was attributed to association with MDR and increased virulence, although the latter was not supported in some studies. Low prevalent ancient/ancestral Beijing sublineage received much less attention. These strains in Japan were isolated mainly from the older population while increasingly circulating modern Beijing strains were more frequently recovered from the younger population hence the conclusion about reduced transmission capacity of ancient Beijing sublineage [19]. On the other hand, a San Francisco study demonstrated an increased transmission (assessed by VNTR-based clustering) of the ancient Beijing strains [20]. A study in Brazil and Mozambique showed that ancient Beijing sublineage displayed intermediate or low levels of virulence in mice model [10]. However, ancient Beijing sublineage is heterogeneous and neither of these strains from Brazil and Mozambique was related to the strains from Russia (Figure S5).

**Figure 7.**
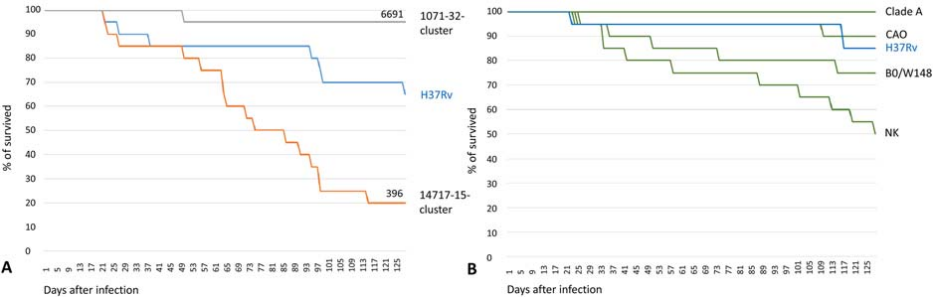
Comparison of survival of mice after infection with *M. tuberculosis* strains within 125 days p.i. in the similarly designed studies. The same strain H37Rv was used as reference. (a) ancient Beijing sublineage (this study). Strain 6691 belongs to 1071-32- cluster (RD181 deleted), strain 396 belongs to 14717-15-cluster (RD181 intact). (b) modern Beijing sublineage (adapted from Bespyatykh et al. [14]). B0/W148 strain is also named Russian epidemic or successful cluster. CladeA, CAO, NK strains belong to Central Asian Russian clade. The green colour is used to show that all clinical isolates in this panel belong to the modern sublineage of the Beijing genotype. Phylogenetic position of these clades and clusters is shown in Figure S1.

Keeping the issue of comparability in mind, we looked at our data in the light of the previous study of the modern Beijing sublineage in Russia [14]. Mouse studies are recognized to be extremely time-consuming and cannot target many strains. Thus, an informed use of the previous results, whenever possible, is helpful. The above-cited study [14] was performed in the same laboratory of experimental tuberculosis under the same design and using the same strain H37Rv. The mice were obtained from the same animal nursery and both studies were carried out in the same period of the year (January-July 2018 and 2020). While the experiments were not identical and a caution in interpretation is needed, nonetheless we believe the survival curves in two panels of Figure 7 are comparable and demonstrate the objective difference between the studied clinical isolates of the modern and ancient Beijing sublineages.

Thus, strain 6691 from Omsk (Beijing early ancient 2, 1071-32 cluster) was among the least virulent Beijing strains, and similar to the low virulent strain of Clade A that is not particularly associated with MDR in Russia [21] (Figure 7). In contrast, Buryat strain 396 (Beijing early ancient 1, 14717-15-cluster) was the most lethal even compared to the notorious MDR epidemic clones B0/W148 and CAO (Figure 7).

The importance of using comparable models of virulence is exemplified by studies of the other important Beijing subtype, the CAO strain [14,22]. CAO stands for Central Asian Outbreak that is a particular cluster within a larger and more heterogeneous Central Asian/Russian clade of the Beijing genotype modern sublineage (Figure S1). CAO is MDR-associated and epidemic in the former Soviet Central Asia but spread also in Russia. In the first study, CAO was among the least lethal strains with 90% survived animals after 125 days [14]. This is drastically different from the second study [22] where all CAO-infected mice succumbed within 47 days. This could be due to different study design and used animals, although could be partly explained by that fact that CAO strains originated from different parts of Russia (St. Petersburg in North and Rostov in South). For example, laboratory strains H37Rv used in different murine experiments also differ in their virulence from low to moderate ([10] and references therein) due to the known genetic diversity of the H37Rv strains in different laboratories and even evolving over time.

The main limitation of the mice experiments, as well as other virulence challenge studies comparing different phylogenetic groups of *M. tuberculosis*, is the relatively small number of strains that can be investigated in each experiment [23,24]. In contrast, in vitro experiment is easier to perform and we compared isolates of the two clusters in their growth rate as an indicator of bacterial fitness. Seven isolates per cluster were analysed. When these results are compared to those obtained in the animal experiment, it is interesting to note that strain 396 had a very short lag-phase 55.9 h and log-phase 142.7 h compared to strain 6691 (69.8 and 160.9 h, respectively; Table S2) in the vitro growth experiment. Altogether shorter time of the lag-phase implies a shorter time for adaptation for 14717-15-cluster compared to 1071-32-cluster: 81.7 vs 116.5. This seems to corroborate with the strongest correlation between high bacterial load and lung damage induced by strain 396 (Figure S4).

During an *M. tuberculosis* infection in mice, bacterial load in lungs is rapidly increased within the first 3 weeks, followed by either stabilization or slow increase over a long period of several months suggesting an inhibitory effect of acquired immunity [1,10]. In our study, the difference in bacterial load in lungs between three strains was most pronounced at day 21 p.i. (Figure 4). Bacterial load showed a longer increase in strain 396 which means that the inhibitory effect of the acquired immunity was established at the later period, i.e. it took more time for mice to establish immunity to contain this strain.

### Transmissibility and virulence

Previous studies suggested that transmissible strains evolve through increasing their virulence and vice verse. Recently evolved and actively transmitted strains of the modern Beijing sublineage were more virulent in mice than the ancient strains whereas the latter were isolated from unique cases or groups of immunocompromised patients [10,23]. A study of the representative Beijing strains showed that susceptible BALB/c mice infected with the highly transmitted Beijing strains began showing mortality 3 weeks post-infection and all had died by 5 weeks, suggesting high virulence phenotypes. In contrast, >80% of mice infected with the non-transmitted strains survived 4 months post-infection, suggesting low virulence phenotypes [23]. The above findings concern modern Beijing sublineage but a study in San Francisco demonstrated that some of the ancient Beijing sublineage strains exhibited levels of virulence in the guinea pig model similar to or even higher than those of the modern Beijing sublineage strains [24] and appeared more transmissible based on VNTR clustering [20]. Thus irrespective of sublineage, more virulent strains appeared more transmissible. On the other hand, in Japan, the recently transmitted TB cases were more frequently caused by less prevalent but emerging strains of the modern sublineage, whereas isolates of the historically dominant ancient sublineage were associated with cases of disease reactivation in older patients and not with the ongoing transmission [19].

Thus, the higher virulence of certain *M. tuberculosis* strains was linked to their increased transmissibility in several studies. In contrast, we demonstrate that there is a specific and more complex situation exemplified by the hypervirulent and highly lethal strain 396. It belongs to the Beijing 14717-15-cluster that accounts for non-negligible 16% of the total *M. tuberculosis* population in Buryatia, Far East. However only very rare isolates of this cluster were described in other Russian regions – 11/423 (2.6%) in Omsk and single isolate per region in Irkutsk, Sakhalin, Kemerovo – all are in the Asian part of Russia ([11] and references therein) (Figure S2). A closer look at human migration data to/from Buryatia reveals that the region has lost 402700 people since the beginning of the 1990s (while its population is 985900) as a result of the *permanent* economic migration outflow to the more developed regions [25]. This situation highlights a clear opportunity for this strain to be spread outside Buryatia through massive population outflow. In reality, it is almost invisible elsewhere, except for rare sporadic isolates across Siberia and the Far East, which however did not grow to the clusters (Figure S2). Accordingly, this hypervirulent/hyperlethal strain is endemically prevalent in Buryatia nonetheless it is not transmitted elsewhere and we term it *conditionally* transmissible. This is in line with observations from the other study of the Russian Beijing strains in Spain [26]. Two XDR strains Beijing B0/W148 and Beijing CAO were isolated in Almeria in 2015 from Russian immigrants to Spain, female commercial sex workers, who lived in Almería for 2 and 3 years. However subsequent retro- and prospective analysis of all new cases did not reveal secondary cases and lack of transmission thereof ([26] and Dario Garcia de Viedma, pers. comm.).

On the other hand, the other studied strain 6691 from Omsk belongs to the MDR/XDR 1071-32-cluster relatively widespread across different FSU countries but at low prevalence and relatively visible only in Omsk, Western Siberia (7%) (Figure S2). This situation follows a traditional theory that multiple drug resistance mutations reduce fitness and virulence and is in line with the above-cited articles that describe the low virulent strains that are low transmitted.

It may be that an increased transmissibility of a strain may be also linked to the propensity and ability to form latent infections. Tuberculosis can be a very slow disease taking decades to form clusters. It is conceivable that some less virulent strains may also transmit but be more likely to form latent infection, and thus also lead to clusters but over a much longer time-frame. This may depend on the phylogenetic background of a strain and the emergence of clusters from different clades may occur over different time-frames. This could also partly explain different findings and conclusions with regard to the (non)correlation between virulence and transmissibility.

## Conclusion

To conclude, both studied MDR strains 396 and 6691 of the ancient Beijing sublineage from Russia were capable of causing tuberculosis with a predominant lung lesion after intravenous infection of mice. Mortality rates of infected mice correlated with the severity of pathological changes in the lung tissue, as well as the bacterial load in the lungs and spleen.

The virulent properties of the clinical strain 396 were most remarkably pronounced. This was demonstrated by high and rapid lethality manifested by the lethality dynamics (massive death of mice), and 100% lethality recorded earlier in animals infected with this strain. In agreement with these data, the bacterial load in the lungs was significantly higher in the strain 396-infected group in all periods of observation. Furthermore, the highest values of other parameters and almost all characteristics concerning pathological changes in the lungs, were observed earlier in the strain 396-infected mice than in other mice. This strain demonstrated the highest lethality among all tested Russian strains, including notorious epidemic clusters B0/W148 and CAO.

The virulence of the other studied clinical strain 6691 strain was significantly lower compared to the reference strain H37Rv and strain 396, which was concordantly demonstrated by all indicators of the severity of the disease. Strain 6691 belongs to the Beijing 1071-32-cluster widespread across different FSU countries but at low prevalence and is relatively visible only in Omsk, Western Siberia (7%). This situation follows a traditional assumption that multiple drug resistance mutations reduce fitness and virulence. In contrast, the highly lethal and hypervirulent strain 396 represents an intriguing Beijing 14717-15-cluster predominant only in Buryatia, Far East (16%), sporadically found beyond it, but not forming clusters of transmission. This specific case does not fit a theory of the highly virulent and highly transmitted strain. We term this cluster 14717-15 conditionally transmissible as it is endemically prevalent only in one location. The reasons may lie in the particular interplay of the human immune system and the genetic background of this strain, and further in-depth study is warranted.

Different modes of adaptation developed by different *M. tuberculosis* variants result in different dispersal patterns, e.g. extremely virulent and lethal strains are less widespread (more limited in their dispersal) although this can be a result of the long-term adaptation to the local human population. At the same time, the mechanisms by which strains implement different levels of virulence and transmissibility have not been fully deciphered. In this regard, further comparative genomic, proteomic, and transcriptomic studies along with human WGS/WES GWAS analysis are needed to comprehensively explain the mechanisms underlying the observed differences in virulence of *M. tuberculosis* strains. Furthermore, while the aim of this study was to analyse the biological properties of these drug-resistant clusters in the model experiment, a large-scale population-based study is required to analyse their clinical significance.

## Supplementary Material

Supplementary_data_EMI.docx

## Data Availability

The data that support the findings of this study are available from the corresponding author, I.M., upon request.
