# Supplementary material for: Extremely lethal and hypervirulent Mycobacterium tuberculosis strain cluster emerging in Far East, Russia
Source: Emerg Microbes Infect. 2021 Aug 22;10(1):1691–701. doi: 10.1080/22221751.2021.1967704 (PMC8381949; doi:10.1080/22221751.2021.1967704)
Supplement: Supplementary_data_EMI.docx [file TEMI_A_1967704_SM8158.docx]

Table S1. Assessment of the index of lung damage

| *Exudative changes and score* | *Productive foci and score* |
| --- | --- |
| aerated lungs – 0 | single submiliary foci – 0.5 |
| single airless foci – 0.25 | multiple submiliary foci (≤ 20) – 1.0 |
| Half-airless lungs – 0.5 | multiple submiliary foci (> 20) –1.5 |
| Two/third airless lungs – 0.75 | single miliary foci –1.75 |
| Airless lungs – 1.0 | multiple merging submiliary and single miliary foci– 2.0 |
|  | multiple miliary foci (≤10) –2.25 |
|  | multiple merging miliary – 2.75 |
|  | appearance of small caseous necrotic foci –3.0 |
|  | extensive caseosis –4.0 |
|  | complete lung damage – 5.0 |

Table S2. Growth characteristics of the studied *M. tuberculosis* strains

| Strain | Cluster (Mlva-based) | Origin | Lag-phase, hs | Lag + Log phases, hs | Time of maximum hourly increase of fluorescence compared to the previous time point, hs | Log-phase, hs | Growth rate coefficient r |
| --- | --- | --- | --- | --- | --- | --- | --- |
| 361 | 14717-15 | Buryatia, Far East | 85.0 | 251.2 | 158.0 | 166.2 | 0.03462 |
| 396 | 14717-15 | Buryatia, Far East | 55.9 | 198.6 | 105.0 | 142.7 | 0.04761 |
| 362 | 14717-15 | Buryatia, Far East | 85.4 | 258.1 | 160.0 | 172.7 | 0.03202 |
| 2690 | 14717-15 | Vologda, northwestern Russia | 52.7 | 206.7 | 97.9 | 154.0 | 0.05078 |
| 380 | 14717-15 | Buryatia, Far East | 83.9 | 397.6 | 136.5 | 313.7 | 0.04427 |
| 390 | 14717-15 | Buryatia, Far East | 131.0 | 376.7 | 200.3 | 245.7 | 0.03461 |
| 8310 | 14717-15 | Omsk, western Siberia | 78.2 | 276.2 | 170.5 | 198.0 | 0.03100 |
| 98 | 1071-32 | Komi, northwestern Russia | 115.2 | 326.7 | 197.7 | 211.4 | 0.02773 |
| 47842 | 1071-32 | Omsk, western Siberia | 145.3 | 381.8 | 217.0 | 236.4 | 0.03139 |
| 10096 | 1071-32 | Omsk, western Siberia | 108.8 | 305.6 | 188.2 | 196.8 | 0.02915 |
| 6691 | 1071-32 | Omsk, western Siberia | 69.8 | 230.7 | 130.5 | 160.9 | 0.04086 |
| 22109 | 1071-32 | Omsk, western Siberia | 141.1 | 329.9 | 218.6 | 188.8 | 0.03013 |
| 7435 | 1071-32 | Omsk, western Siberia | 130.8 | 305.1 | 202.5 | 174.3 | 0.03356 |
| 6702 | 1071-32 | Omsk, western Siberia | 104.3 | 256.2 | 164.1 | 151.9 | 0.04497 |

The average values of the data obtained in three experiments are given for each strain.

**Figure S1.** Simplified evolutionary scheme of *M. tuberculosis* Beijing genotype with a focus on the genotypes and sublineages analyzed or cited in this article.

**Figure S2**. Geographical distribution of the two studied clusters of the *M. tuberculosis* ancient Beijing sublineage in different locations in northern Eurasia. Locations of origin of the two strains included in the virulence mouse model study are in bold: Omsk, western Siberia (strain 6691) and Buryatia, Far East (strain 396). Reproduced with modification from Mokrousov et al. 2019. Copyright Elsevier Ltd.

**Fig. S3.** The study design graph.

**Fig. S4.** Correlation between bacterial load and lung pathology scores for three studied

*M. tuberculosis* strains in the days 3 to 28 p.i.

Correlation coefficient is shown in the upper right part of the each graph.


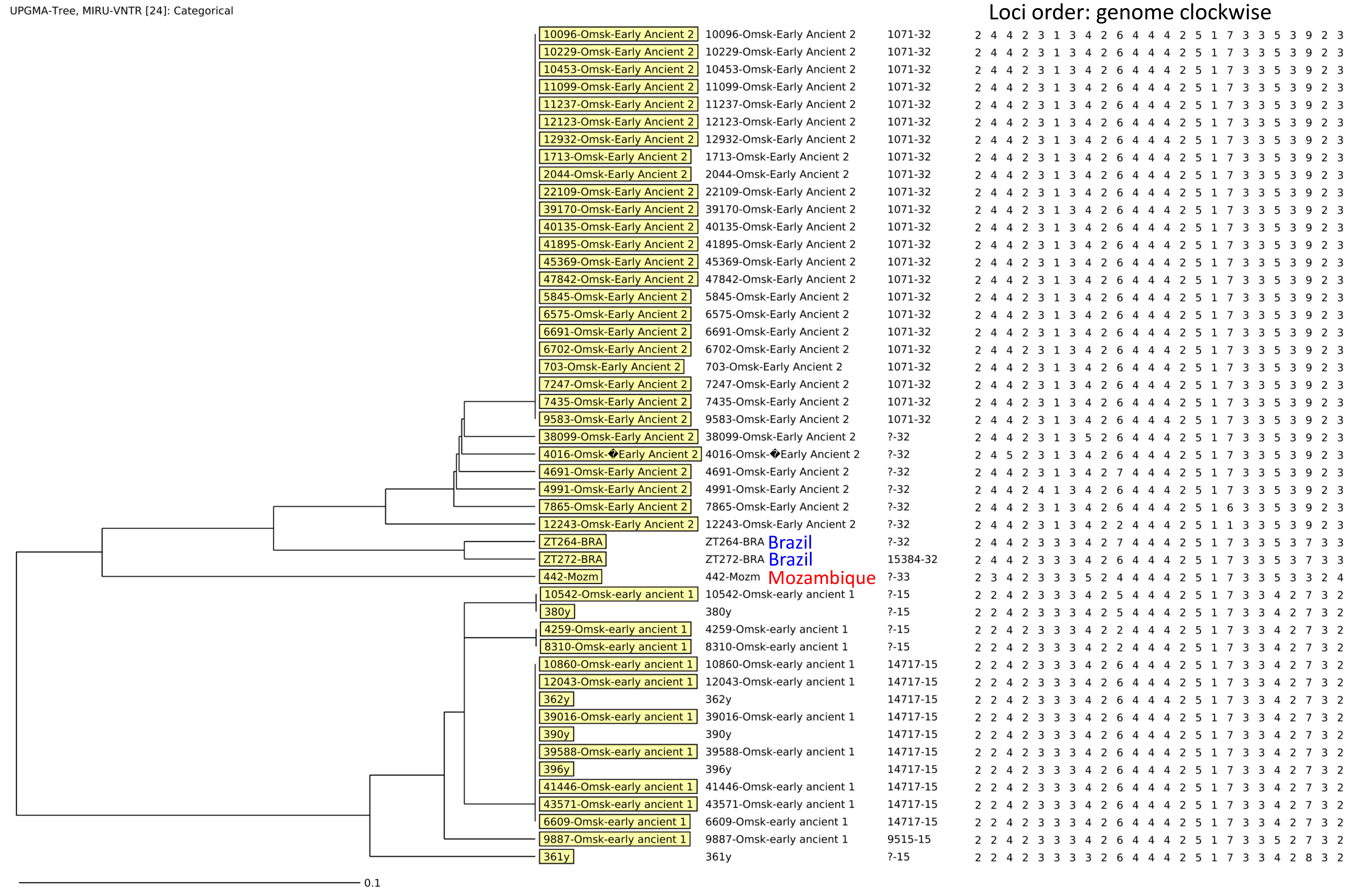


**Figure S5.** 24-VNTR dendrogram of ancient Beijing *M. tuberculosis* strains from Russia, Brazil and Mozambique.

Based on genotyping data from this study, our previous study (Mokrousov et al., 2019) and Brazil and Mozambique study (Ribeiro et al., 2014). According to the terminology of Mokrousov et al. (2020), Russian strain 396 (VNTR 14717-15 mainly from Buryatia, Far East) belongs to deeply rooted early ancient 1 sublineage (*mutT4*-48 wt, RD181 intact) while strain 6691 (VNTR 1071-32 mainly from West Siberia) and strain from Mozambique belonged to early ancient sublineage 2 (*mutT4*-48 wt, RD181 del.). Finally, two strains from Brazil belonged to classical ancient Beijing (*mutT4*-48 mut). All these strains had wild type allele of muT2-58 which a key marker of the ancient Beijing on the whole (**Fig. S1**).
